# Dynamic active telepathology over National Health Laboratory service network, South Africa: feasibility study using Nikon Coolscope

**DOI:** 10.1186/1746-1596-3-S1-S3

**Published:** 2008-07-15

**Authors:** Lech Banach, Andrzej Stepien, Johann Schneider, Elizabeth Wichrzycka-Lancaster

**Affiliations:** 1 NHLS and Department of Anatomical Pathology, Walter Sisulu University, Mthatha, South Africa; 2 NHLS and Department of Anatomical Pathology, University of Stellenbosch, Cape Town, South Africa; 3 NHLS and Department of Anatomical Pathology, University of Limpopo, Pretoria, South Africa

## Abstract

Telepathology recently entered a new era with the introduction of digital microscopes combined with Internet technology. The microscope allows viewing real time of whole slide (macro) as well as different chosen fields in four different magnifications. Three Nikon Coolscope were installed in NHLS laboratories in Mthatha, East London and Port Elizabeth. All these microscopes are connected to NHLS server allowing real time viewing of the full slide at any time of the day using Internet browser. Viewing is possible from any PC connected to NHLS Intranet. The challenge was to be able to view slides from other than NHLS computers due to NHLS IT Department network security measures. This was solved by installing NHLS Virtual Private Network server. About 60 cases were viewed by pathologists in Cape Town (Stellenbosh University) and Pretoria (MEDUNSA). All users assessed the system as a helpful tool allowing easy access to cases needing consultation or second opinion. The quality of images was very good. Our experience with Nikon Coolscope is positive. It is an excellent tool for remote small histopathology departments lacking specialists in such areas as dermatopathology, oncology, and haematopathology. Further studies are needed especially in the scope of full utilization of the microscopes installed and impact on laboratory services.

## Introduction

South Africa experiences huge social and health services challenges due to the HIV/AIDS epidemic (about 5 million infected) and shortage of medical staff, mostly specialists. This includes pathology services as many pathologists left the country for greener pasture or retired. The pathology pattern seen in histopathology laboratories also changed due to increased HIV/AIDS associated pathology (neoplasms and skin lesions). Referral of cases from peripheral pathology labs to specialized centers is costly and time consuming. Many of these cases could be diagnosed remotely using telepathology. The introduction of digital microscopes such as Nikon Coolscope [1,2] revolutionized dynamic active telepathology as these are all-in-one instruments combining ordinary microscope with digital camera and network function with dimension of ordinary PC. The Coolscope microscope could be connected to the LAN or WAN and controlled by the remote user over Coolscope graphical user interface on standard browser. Images are presented as live video, while a motorized stage permits control from a remote site. Objective selection, focusing and other functions can likewise be managed by remote operator. Coolscope is used by some centers: Department of Pathology, Basel University, Marcy ship Anastasis with connection to UK and a missionary hospital in Zambia. We describe our project of linking pathology laboratories over NHLS network for telepathology services in Eastern Cape Province of South Africa.

## Settings/method

**Figure 1:**
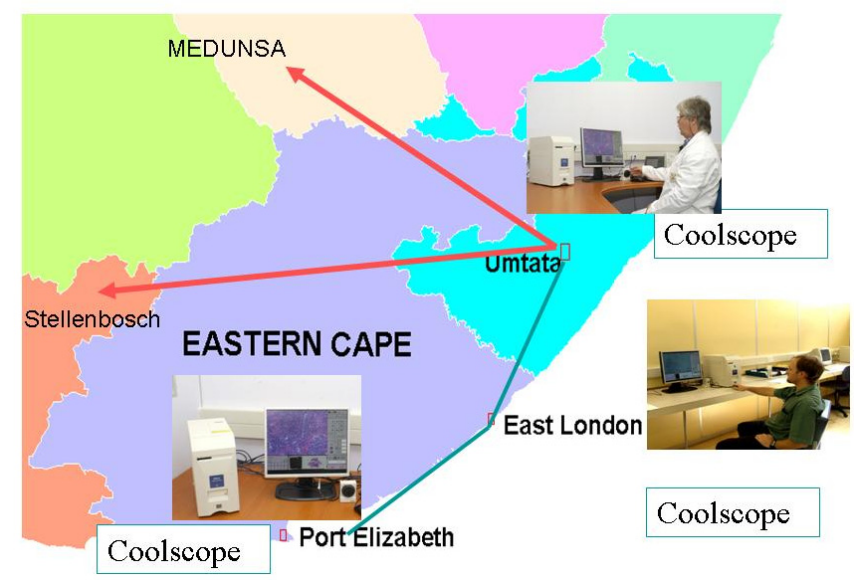
Eastern Cape tertiary NHLS laboratories with Coolscopes installed and connected via LAN.

Three Coolscopes were installed in NHLS laboratories in Mthatha, East London and Port Elisabeth and connected via LAN allowing remote control of the microscope from any PC on NHLS network (Figure 1). These laboratories are run by only few pathologists, without access to full immunohistochemical studies, which makes final diagnosis difficult.

**Figure 2:**
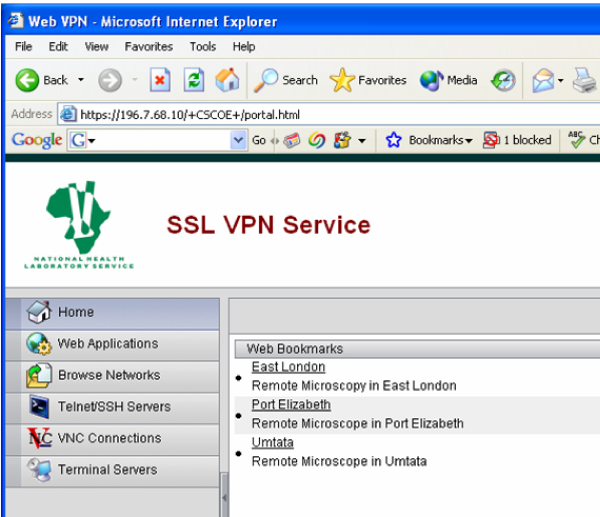
NHLS VPN Web Page with connectivity to Coolscopes.

**Figure 3:**
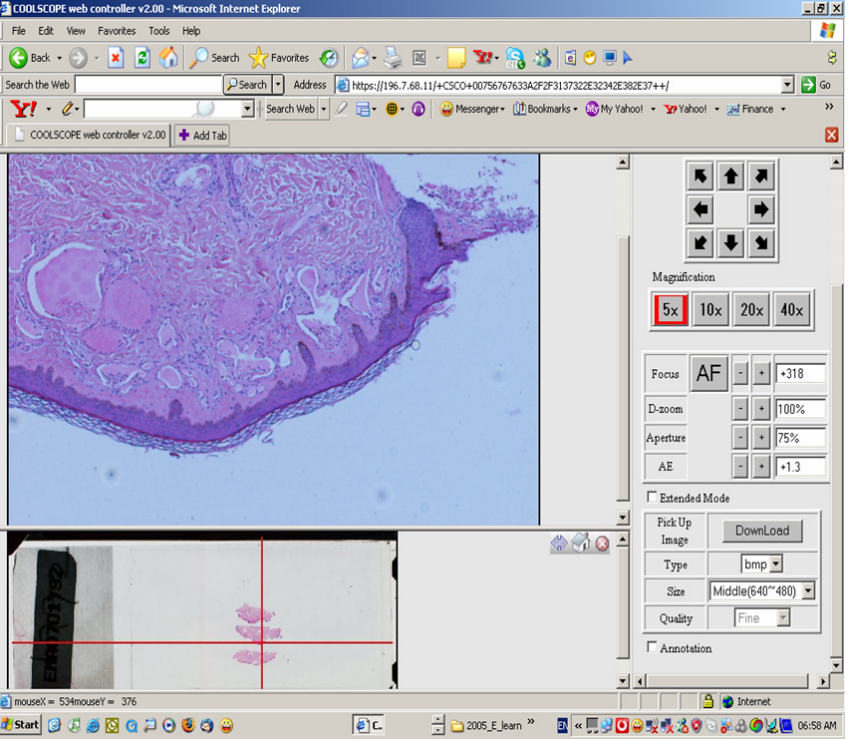
Coolscope Interface for remote control.

Due to security measures on NHLS LAN it was not possible to connect to Coolscopes outside LAN. Virtual Private Network Server was installed to allow connection from any PC at any time and from any site. NHLS VPN server uses Secure Socket Level (SSL) technology with security level compared to this used in e-banking or e-commerce (Figure 2). Example of web page with Coolscope interface is seen on Figure 3.

## Results

About 60 sessions were performed using Coolscope in Mthatha: 30 sessions were performed using LAN and 30 using virtual private network. The best results were obtained using Coolscope over LAN (3–6 sec load time). Slower control was experienced using VIP from home or university network. This was mostly due to communication problems. The best connectivity was via wireless 3G, which offers up to 3.6 Mbts speed. The system was used mostly for current cases in dermatopathology, malignant neoplasia and haematopathology.

## Discussion

Online dynamic active (live) telepathology is mostly used for primary tissue based diagnosis, such as frozen section diagnosis, and also for second opinion or consultation, e.g. in dermatopathology, neuropathology and cytology [3-6]. It has potential for quality control as well as educational programs such as correlation of cytology and histopathology specimens, slide seminars, clinico-pathological meetings and discussion groups.

## Conclusion

Users of Coolscopes in this study were quite satisfied with the system installed. However detailed evaluation is needed especially in respect of impact on laboratory performance and usage of Coolscope in teleducation.
